# Autophagy is Involved in Neuroprotective Effect of Alpha7 Nicotinic Acetylcholine Receptor on Ischemic Stroke

**DOI:** 10.3389/fphar.2021.676589

**Published:** 2021-04-29

**Authors:** Zhe-Qi Xu, Jing-Jing Zhang, Ni Kong, Guang-Yu Zhang, Ping Ke, Ting Han, Ding-Feng Su, Chong Liu

**Affiliations:** Department of Pharmacy, Second Military Medical University, Shanghai, China

**Keywords:** α7 nicotinic acetylcholine receptor, autophagy, ischemic stroke, neuroprotection, apoptosis

## Abstract

The α7 nicotinic acetylcholine receptor (α7nAChR) belongs to the superfamily of cys loop cationic ligand-gated channels, which consists of homogeneous α7 subunits. Although our lab found that activation of α7nAChR could alleviate ischemic stroke, the mechanism is still unknown. Herein, we explored whether autophagy is involved in the neuroprotective effect mediated by α7nAChR in ischemic stroke. Transient middle cerebral artery occlusion (tMCAO) and oxygen and glucose deprivation (OGD/R) exposure were applied to *in vivo* and *in vitro* models of ischemic stroke, respectively. Neurological deficit score and infarct volume were used to evaluate outcomes of tMCAO in the *in vivo* study. Autophagy-related proteins were detected by Western blot, and autophagy flux was detected by using tandem fluorescent mRFP-GFP-LC3 lentivirus. At 24 h after tMCAO, α7nAChR knockout mice showed worse neurological function and larger infarct volume than wild-type mice. PNU282987, an α7nAChR agonist, protected against OGD/R-induced neuronal injury, enhanced autophagy, and promoted autophagy flux. However, the beneficial effects of PNU282987 were eliminated by 3-methyladenine (3-MA), an autophagy inhibitor. Moreover, we found that PNU282987 treatment could activate the AMPK-mTOR-p70S6K signaling pathway in the *in vitro* study, while the effect was attenuated by compound C, an AMPK inhibitor. Our results demonstrated that the beneficial effect on neuronal survival via activation of α7nAChR was associated with enhanced autophagy, and the AMPK-mTOR-p70S6K signaling pathway was involved in α7nAChR activation–mediated neuroprotection.

## Introduction

Ischemic stroke is a clinical condition characterized by sensory dysfunction, motor dysfunction, cognitive dysfunction, or language dysfunction due to lack of blood flow (Balami et al., 2013). It is one of the most common causes of death and a leading cause of disability worldwide, which leads to a heavy burden for patients’ families and society (Horner, 1998; Global Burden of Disease Study 2013 Collaborators 2015). After the blockage of blood vessels by thrombotic or embolic occlusion, ischemia can lead to a series of devastating cascade reactions in the brain tissue, such as generation of reactive oxygen species, calcium overload, inflammation, and apoptosis, which can result in neuronal injury and death (Tang et al., 2015; Chamorro et al., 2016; Radak et al., 2017; Zhao et al., 2018). Although a complicated mechanism of pathophysiology following ischemic stroke has been uncovered by scientists in recent decades, the exact mechanism has not been fully understood, and the need to discover a new therapeutic target is still urgent.

The α7 nicotinic acetylcholine receptor (α7nAChR) belongs to the superfamily of cys loop cationic ligand-gated channels, which consists of homogeneous α7 subunits (Dineley et al., 2015). It is widely expressed throughout brain tissue including both neurons and nonneuronal cells (Freedman et al., 2000; Wang et al., 2013; Dineley et al., 2015; Egea et al., 2015). Previously, our studies found that the activation of α7nAChR on microglia could lead to anti-inflammatory effect, which plays an alleviative role in experimental autoimmune encephalomyelitis (EAE) and ischemic stroke mice (Qian et al., 2015; Shao et al., 2017). Besides, studies from other labs also found that activation of α7nAChR is beneficial to many central nervous system diseases such as Alzheimer’s disease, schizophrenia, and stroke (Han et al., 2014a; Dineley et al., 2015; Nakaizumi et al., 2018). However, the mechanism of the protective effect of α7nAChR on the central nervous system diseases has not been fully understood.

Autophagy is a cellular process to degrade and recycle misfolded or long-lived proteins and damaged organelles (Moulis and Vindis, 2018). There are three different forms of autophagy including macroautophagy, microautophagy, and chaperone-mediated autophagy, out of which macroautophagy is the main pathway (Wang et al., 2017). The autophagy flux involves the double membrane elongating to the autophagosome to engulf organelles and proteins, the autophagosome fusing with the lysosome to form the autolysosome, and then the organelles and proteins being degraded (Moulis and Vindis, 2017). It is indisputable that ischemia can enhance autophagy (Wen et al., 2008). Previous studies showed that autophagy is related to many central nervous system diseases such as Alzheimer’s disease, stroke, and multiple sclerosis (Lipinski et al., 2010; Dang et al., 2014; Wei et al., 2016; Kerr et al., 2017). However, whether autophagy plays a protective role or a detrimental role in ischemic stroke is still unclear (Chen et al., 2014; Li et al., 2015; Dai et al., 2017; Song et al., 2017; Wang et al., 2018).

In this study, we investigated the role of autophagy in ischemic stroke and whether it is involved in the protective effect of α7nAChR on ischemic stroke and how that works.

## Methods

### Animal Care and Use

C57BL/6 mice were purchased from Shanghai Super B&K Laboratory Animals Co., Ltd. (Shanghai, China). α7nAChR knockout mice (B6.129S7-Chrna7^tm1Bay^/J, Stock No:003232) were purchased from the Jaxson Laboratory (Bar Harbor, ME, United States). All animals were taken good care of according to the National Institute of Health Guide for the Care and Use of Laboratory Animals, and the experiments were approved by the Second Military Medical University Ethics Committee for Animals. Operations were performed in accordance with guidelines. The mice were housed in a 12 h light/dark cycle with free access to food and water at 20 ± 4°C temperature and 10% humidity.

### Transient Middle Cerebral Artery Occlusion

The transient middle cerebral artery occlusion (tMCAO) operation was induced as previously reported (Liu et al., 2012a; Kraft et al., 2013). In brief, the mice were anesthetized with 1% pentobarbital sodium (0.1 g/kg, i.p.). Cerebral focal ischemia was produced by intraluminal occlusion of the right middle cerebral artery using a silicone-coated nylon monofilament (diameter, 0.23 ± 0.02 mm). Throughout the surgery, the mice were put on a temperature controller pad (Nanjing Xin Xiao Yuan Biotech, Nanjing, China) to maintain the core body temperature at 37°C. After 2 h of MCAO, the occluding filament was withdrawn for reperfusion. The extent of ischemia was determined by cerebral blood flow using a laser Doppler Blood Flow Meter (MNP110XP, ML191, AD Instruments, Australia), and only those mice which showed a 70% reduction of blood flow were used for further experiments. For the mice in sham groups, surgery was performed in the same way as in the tMCAO group, except for insertion of filament. 24 h after tMCAO or sham surgery, the mice were scored for neurological deficit and killed for further experiments.

### Neurological Deficit Scoring

To determine the neurological function after tMCAO, a five point scale was used as previously reported (Guo et al., 2013). In brief, 0 point is for mouse with normal motion; 1 point is for mouse with contralateral forearm bending to the torso when lifting its tail; 2 point is for mouse which is circling to the contralateral side but has normal posture at rest; 3 point is for mouse which is circling to the contralateral side and has abnormal posture at rest; and 4 point is for mouse without autonomous movement.

### Infarct Volume Measurement

Infarct volume was determined at 24 h after tMCAO surgery (Jung et al., 2016; Justicia et al., 2017). Each brain was drawn and cut into five slides which were 2 mm thick by a brain-cutting matrix. The slides were stained with 1% 2,3,5-triphenyltetrazolium chloride (TTC) solution (Sinopharm Chemical Reagent Co., Ltd., Shanghai, China) at 37°C for 30 min in a dark room. The slides were dried and photographed. The infarct volume and contralateral hemispheric volume were determined by using Image J software. A correction for edema was made in each area by multiplying the infarct area by the ratio of the contralateral to the ipsilateral hemisphere. At last, infarct volume was displayed by a percentage rate of infarct volume to contralateral hemispheric volume.

### Culture of Primary Cortical Neurons

Primary cortical neurons were extracted and cultured as previously reported (Yang et al., 2014). Cortical neurons were isolated from the cerebral cortex of E16–E18 mouse embryos. In brief, pregnant mice supplied by Shanghai Super B&K Laboratory Animals Co., Ltd. (Shanghai, China) were euthanized. The cerebral cortex of the embryos was collected, and the meninges were carefully separated from the cerebral cortex and discarded. The cerebral cortex was cut into small pieces and digested with accutase enzyme (Gibco, MA, United States) at 37°C for 10 min. The digestion was stopped by using high glucose DMEM (Gibco, MA, United States) with 20% fetal bovine serum (FBS, Gibco, MA, United States) and pipetting gently to get single cell suspension. Cell suspension was centrifuged at 1,000 rpm for 5 min, and the supernatant was discarded. The cells were resuspended with high glucose DMEM with 20% FBS. After that, the cells were filtered through a 40 μM strainer and centrifuged at 1,000 rpm for 5 min, and the supernatant was discarded. Resuspended cells with high glucose DMEM with 20% FBS were plated into poly-D-lysine–coated 6-well (7 × 10^5^ cell/well) plates or 96-well (4 × 10^4^ cell/well) plates. At 2 days *in vitro* (DIV) after plating, we changed the medium with neural basal medium (Gibco, MA, United States) with 2% B27 supplement and 1% penicillin and streptomycin. At DIV 7, neurons were formed with purity ≥90%, and they can be prepared for further experiments.

### Oxygen and Glucose Deprivation(OGD/R) and Drug Treatment

In brief, we discarded the normal neural basal medium and washed the primary cortical neurons with 1× PBS twice and then changed the medium into glucose-free DMEM medium. Cultured cells were put into a tri-gas CO2 incubator (Thermo Scientific Series II 3110 Water-Jacketed, United States) filled with 94% N_2_, 5% CO_2,_ and 1% ≤ O_2_ at 37°C for 2 h. Then, the culture medium was changed to normal neural basal medium to mimic reperfusion, and the neurons were plated in the normal culture condition (5% CO_2_, 37°C). The total time of oxygen and glucose deprivation/reperfusion (OGD/R/R) was 24 h.

At the beginning of OGD/R condition exposure, neurons were administrated with or without the α7nAChR activator PNU282987 (Sigma-Aldrich, St. Louis, MO, United States), with a dose of 1, 10, and 100 μM for 2 h, respectively. Similarly, primary cortical neurons were treated with or without 10 mM 3-methyladenine (3-MA, autophagy inhibitor) and 5 μM compound C (AMPK inhibitor) during the 2 h OGD/R period in a set of respective experiments.

### Cell Viability and Cytotoxicity Assay

Cell viability and LDH release were determined by using commercial kits (CCK-8 kit; Dojindo, Kamimashiki-gun Kumamoto, Japan) and an LDH assay kit (LDH kit, Dojindo, Kamimashiki-gun Kumamoto, Japan). The experiments were conducted according to the protocols. In brief, primary neurons were plated in 96-well plates (4 × 10^4^ cell/well). At DIV 7, the primary cortical neurons underwent OGD/R condition exposure with or without drug treatment. For the CCK-8 assay, 24 h after OGD/R/R, we added 10% CCK-8 working solution to each well of the plate and incubated the plate for 1 h in the incubator. We measured the absorbance at 450 nm using a microplate reader. For the LDH release assay, 24 h after OGD/R/R, we added 100 μl of the working solution to each well and protected the plate from light and incubated it at room temperature for 30 min. Then, we added 50 μl of the stop solution to each well and measured the absorbance at 490 nm by using a microplate reader.

### TUNEL Staining

After OGD/R condition exposure and a variety of drug treatments, apoptotic rate of primary cortical neurons were detected by using the terminal deoxynucleotidyl transferase–mediated dUTP nick end-labeling (TUNEL) technique (*In Situ* Cell Death Detection Kit, Roche, Mannheim, Germany). In brief, primary cortical neurons were plated in 35-mm dishes with a glass bottom (7 × 10^5^ cell). The cells were washed with 1x PBS twice, fixed by 4% paraformaldehyde for 1 h at room temperature, and then permeabilized with 0.1% Triton X-100 for 2 min on ice. We added 50 μl of TUNEL reaction mixture on the sample and incubated it in a humid dark area at 37°C for 60 min. Nuclei were stained with DAPI (10 μg/ml). The dishes were scanned by using a confocal laser scanning microscope (FluoView FV1000; Olympus, Tokyo, Japan), and apoptotic neurons were counted.

### Autophagy Flux Assay

Primary cortical neurons were plated in 35 mm dishes with a glass bottom (7 × 10^5^ cell) and transfected with tandem fluorescent mRFP-GFP-LC3 lentivirus. Briefly, at DIV 3, after the medium in each dish had been changed with 1 ml neural basal medium with 2% B27 supplement and 1% penicillin and streptomycin, primary cortical neurons were transfected with lentivirus with a multiplicity of infection of 10 for 4 h. Then, we added an additional 1 ml of neural basal medium with 2% B27 supplement and 1% penicillin and streptomycin. One day after transfection, we changed it to 2 ml fresh medium. 72 h after transfection, neurons were exposed with OGD/R for 2 h with or without PNU282987 (100 μM). Autophagosomes (G^+^R^+^) and autolysosomes (G^−^R^+^) within neurons were detected by confocal microscopy (FluoView FV1000; Olympus, Tokyo, Japan). The total number of puncta (>1 μm) per cell was counted.

### Reverse Transcription and Real-Time PCR

The mRNA expression of α7nAChR, Bax, and Bcl-2 was detected by real-time PCR. Total RNA from brain tissue or primary cortical neurons was extracted by using Trizol reagent (Invitrogen, Carlsbad, CA, United States). To obtain cDNA, reverse transcription was conducted with PrimeScript^™^ RT Master Mix (Takara, Otsu, Shiga, Japan). The quantitative PCR was performed using LightCycler quantitative PCR apparatus (Stratagene, Santa Clara, CA, United States). Each sample test contained a final volume of 20 μl—10 μl of 2x SYBR Green Real-Time PCR Master Mix (Roche, Konzern-Hauptsitz, Grenzacherstrasse, Switzerland), 5 μl of cDNA, 1 μl of the forward and reverse primers, and 4 μl of ddH_2_O. All samples were conducted in duplicate. The mRNA expressions were normalized to β-actin. The primers are shown in Table 1.

**TABLE 1:** Primers for RT-PCR.

Gene name	Forward primer	Reverse primer
α7nAChR	CAA​TGG​CAA​CCT​GCT​CTA​CA	AGG​TGC​TCA​TCA​TGT​GTT​GG
Bax	GGC​TGG​ACA​CTG​GAC​TTC​CT	GAT​GGT​CAC​TGT​CTG​CCA​TGT
Bcl-2	CGC​AGA​GAT​GTC​CAG​TCA​GC	CCT​GTT​GAC​GCT​CTC​CAC​AC
β-actin	GTG​CTA​TGT​TGC​TCT​AGA​CTT​CG	ATG​CCA​CAG​GAT​TCC​ATA​CC

### Immunoblotting

Brain tissues or primary cortical neurons were washed with PBS and lysed with cell and tissue lysis buffer supplemented with protease/phosphatase inhibitor cocktail. Protein samples were subjected to SDS-PAGE. Then, the proteins in gels were transferred onto nitrocellulose membranes. After blocking in 5% no-fat dry milk in PBS for 1 h, the membranes were incubated with primary antibodies as follows: Bcl-2 (1:500, Proteintech, Wuhan, Hubei, PRC), Bax (1:2,000, Proteintech, Wuhan, Hubei, PRC), caspase 3 (1:500, Proteintech, Wuhan, Hubei, PRC), β-actin (1:5,000, Proteintech, Wuhan, Hubei, PRC), α7nAChR (1:1,000, Proteintech, Wuhan, Hubei, PRC), Beclin1 (1:500, Proteintech, Wuhan, Hubei, PRC), LC3 (1:300, Proteintech, Wuhan, Hubei, PRC), P62/SQSTM1 (1:1,000, Proteintech, Wuhan, Hubei, PRC), Atg7 (1:1,000, Cell Signaling Technology, Danvers, MA, United States), adenosine 5′-monophosphate–activated protein kinase (AMPK) (1:500, Cell Signaling Technology, Danvers, MA, United States), phosphorylated AMPK antibody (1:500, Cell Signaling Technology, Danvers, MA, United States), mammalian target of rapamycin (mTOR) (1:500, Cell Signaling Technology, Danvers, MA, United States), phosphorylated mTOR (1:500, Cell Signaling Technology, Danvers, MA, United States), P70 ribosomal protein S6 kinase (p70S6K) (1:500, Cell Signaling Technology, Danvers, MA, United States), and phosphorylated p70S6K (1:500, Cell Signaling Technology, Danvers, MA, United States). Then, we washed with PBST and incubated with secondary donkey anti-rabbit or donkey anti-mouse antibodies (1:5,000, LI-COR Biosciences, Lincoln, NE, United States). Images were obtained with the Odyssey Infrared Fluorescence Imaging System.

### Immunofluorescence

Brain tissues were fixed in 4% paraformaldehyde and then embedded in paraffin. Then, 5 μm thick sections of brain tissues were deparaffinized with xylene and blocked with goat serum. For primary cortical neurons, cells were fixed in 4% paraformaldehyde for 15 min, washed with PBS, permeabilized with 0.1% Triton X-100 for 5 min, and blocked with goat serum. Prepared samples were incubated with primary antibodies of rabbit anti-α7nAChR (1:200, Proteintech, Wuhan, Hubei, PRC) with or without mouse anti-MAP2 (1:10,000, Abcam, Cambridge, MA, United States) and rabbit anti-LC3 (1:50, Proteintech, Wuhan, Hubei, PRC) with mouse anti-MAP2. After that, sections and dishes were incubated with double immunofluorescent staining including Alexa-488 and Alexa-647 labeled secondary antibodies (1:500, Invitrogen, United States) for 1 h at room temperature. DAPI (Vector Laboratories, Burlingame, CA, United States) was used to stain the nucleus. The sections and dishes were scanned by using a confocal laser scanning microscope (FluoView FV1000; Olympus, Tokyo, Japan). To perform semi-quantitative statistical analysis, the average fluorescence intensity was detected with Image J software. In brief, we split a single channel of the image and then adjusted the threshold with the default method. After setting measurements, we can get the values.

### Statistical Analysis

Data were analyzed with GraphPad Prism 7 (La Jolla, CA, United States) and presented as mean ± SEM with Student’s t-test for two groups or one-way analysis of variance (ANOVA), followed by Sidak’s multiple comparisons test for more than two groups. Data distribution was assessed with the Kolmogorov–Smirnov test, and all values were normally distributed (Kolmogorov–Smirnov test, *p* > 0.10 for each data set). For grouped analysis of more than two groups, two-way ANOVA followed by Sidak’s multiple comparisons tests were used. *p* value < 0.05 was considered statistically significant.

## Results

### α7nAChR Knockout in Mice Deteriorates Ischemic Stroke

**FIGURE 1:**
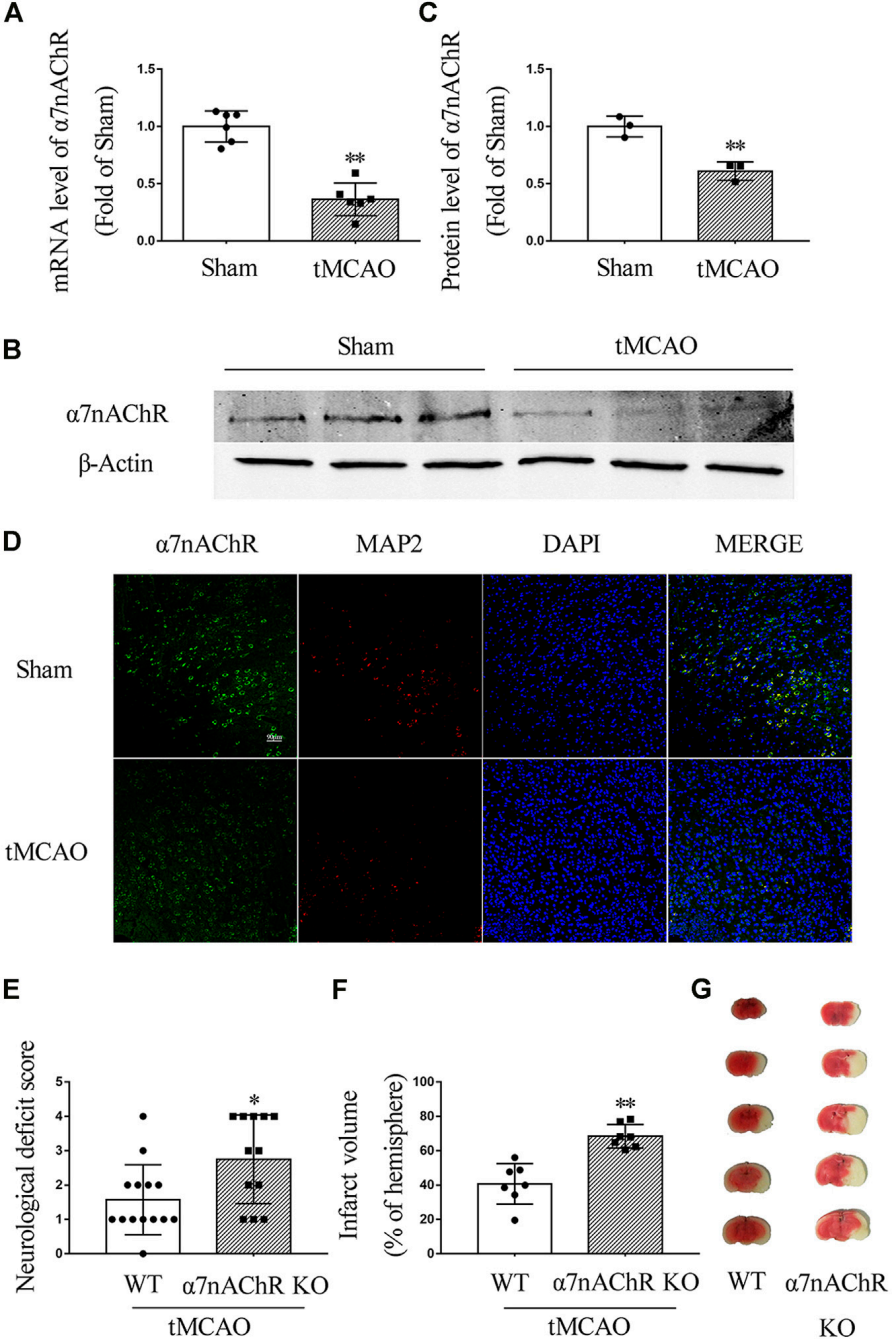
α7nAChR knockout deteriorates ischemic stroke in transient middle cerebral artery occlusion (tMCAO) mice. Mouse brain was collected at 24 h in wild-type (WT) mice and α7nAChR knockout (KO) mice after tMCAO surgery. **(A)** mRNA level in the penumbral zone. *n* = 6 for each group. **p < 0.01 *vs.* Sham. **(B, C)** Protein expression in the penumbral zone. *n* = 3 for each group. **p < 0.01 *vs.* Sham. **(D)** Representative immunofluorescence staining images of α7nAChR, MAP2, and DAPI. Scale bar, 90 μm. **(E)** Neurological deficit score. *p < 0.05 vs*.* WT. *n* = 12–14 for each group. **(F)** Quantification of infarct volume; **p < 0.01 *vs.* WT. *n* = 7 per group. **(G)** Representative image of TTC staining.

To investigate the expression of α7nAChR after ischemic stroke, C57/BL6 mice underwent tMCAO surgery. At 24 h after the surgery, the mRNA level of α7nAChR was determined by real-time PCR, and the protein expression level was determined by Western blot and immunofluorescence. Our results showed that both mRNA and protein levels of α7nAChR in the penumbral zone were significantly decreased at 24 h after ischemic stroke (Figures 1A–D). To evaluate the role of α7nAChR in ischemic stroke, the wild-type (WT) mice and the α7nAChR knockout (α7nAChR KO) mice underwent tMCAO surgery. At 24 h after tMCAO, neurological deficit scores were determined using a 5-point scale, and the brains were collected to determine infarct volume with TTC staining. As shown in Figure 1E, compared with the neurological function of WT mice, the neurological function of α7nAChR KO mice was significantly deteriorated (neurological deficit score: 1.57 ± 0.27 in WT mice *vs.* 2.75 ± 0.37 in α7nAChR KO mice, *p* < 0.05), and the infarct volume in α7nAChR KO mice was increased by 68.16% when compared with that in WT mice (Figures 1F,G).

### Activating α7nAChR Inhibits Apoptosis in Neurons Exposed to OGD/R

**FIGURE 2:**
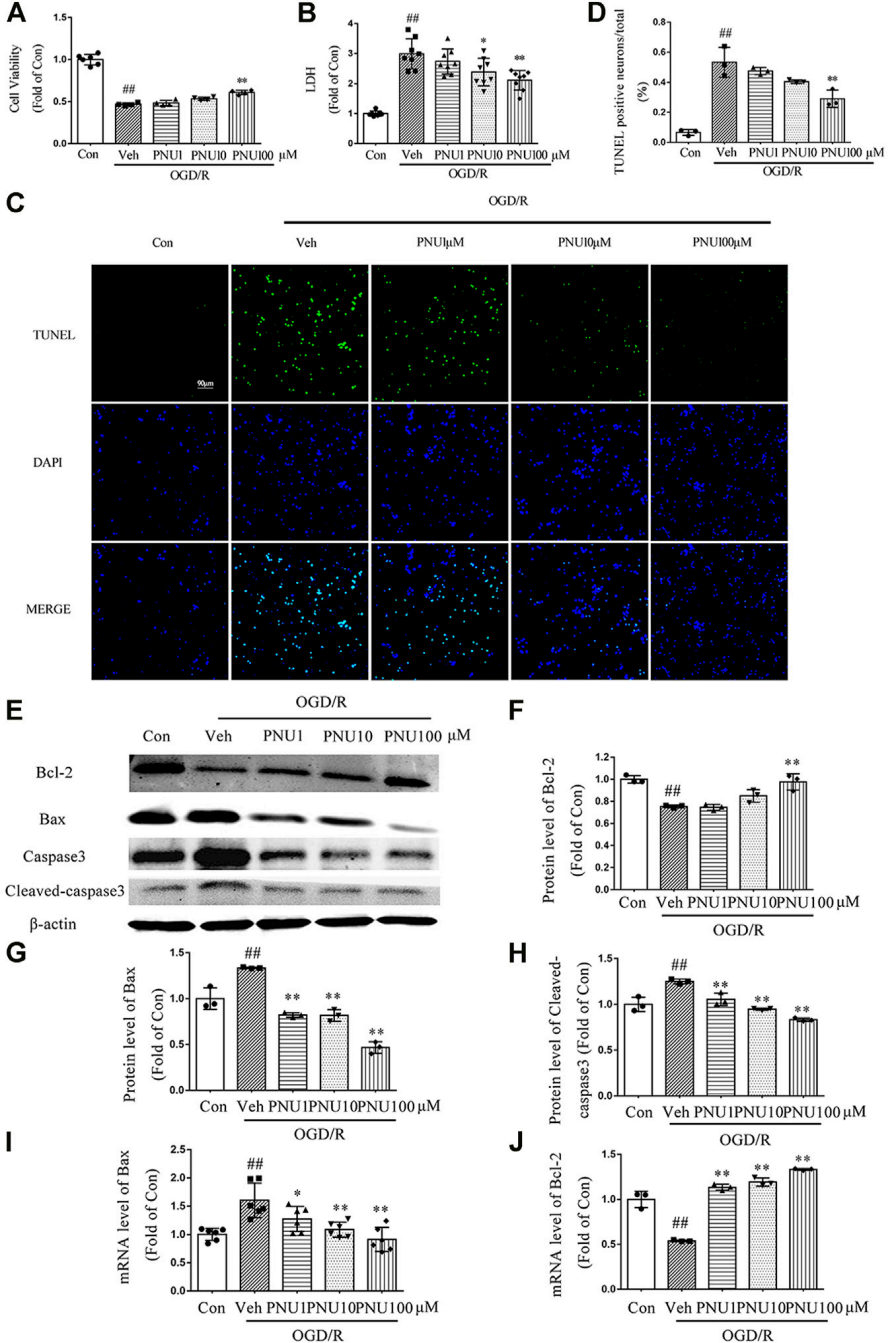
PNU282987 dose-dependently protects neurons from OGD/R injury. Primary cortical neurons were pretreated with vehicle or PNU282987 (1 , 10, and 100 μM) for 2 h before OGD/R exposure. The cell viability and LDH release were examined at 24 h upon OGD/R exposure. **(A)** PNU282987 100 μM could increase the cell viability compared to that in the OGD Veh group. *n* = 4–6 for each group. **(B)** PNU282987 100 μM could decrease the LDH release compared to that in the OGD Veh group. *n* = 8 for each group. **(C)** Representative images of TUNEL. PNU282987 (10 and 100 μM) dose-dependently reduced the number of apoptotic neurons by TUNEL staining. Scale bar, 90 μm. **(D)** Quantification of apoptotic neurons. *n* = 3 for each group **(E–H)** Apoptosis-related proteins including Bcl-2, Bax, caspase 3, and cleaved caspase 3 were detected by Western blot. *n* = 3 for each group. **(I, J)** Apoptosis-related mRNA including Bcl-2 and Bax were detected by real-time PCR. *n* = 3–6 for each group. ^##^ *p* < 0.01 *vs.* con; *p < 0.05 vs*.* OGD/R veh; and **p < 0.01 *vs.* OGD/R veh.

To determine the effect of α7nAChR on viability and death of neurons after OGD/R, PNU282987, a selective agonist of α7nAChR, was used with doses of 1, 10, and 100 μM, respectively. We found that OGD/R induced a significant decrease of neuron viability, while PNU282987 dose-dependently attenuated this effect (Figure 2A). PNU282987 treatment dose-dependently inhibited the increase of LDH release induced by OGD/R exposure (Figure 2B). As shown in Figures 2C,D, PNU282987 could dose-dependently decrease TUNEL positive neurons after OGD/R exposure. Furthermore, OGD/R exposure decreased the expression of Bcl-2 and increased the expression of Bax and cleaved-caspase 3, while PNU282987 treatment significantly inhibited these effects induced by OGD/R (Figures 2E–H). The mRNA levels of Bcl-2 and Bax were consistent with the protein results (Figures 2I,J). These results demonstrated that activation of α7nAChR by PNU282987 could dose-dependently inhibit neuronal apoptosis and protect neurons against OGD/R-induced injury.

### α7nAChR Can Regulate Autophagy in Ischemic Stroke Mice

**FIGURE 3:**
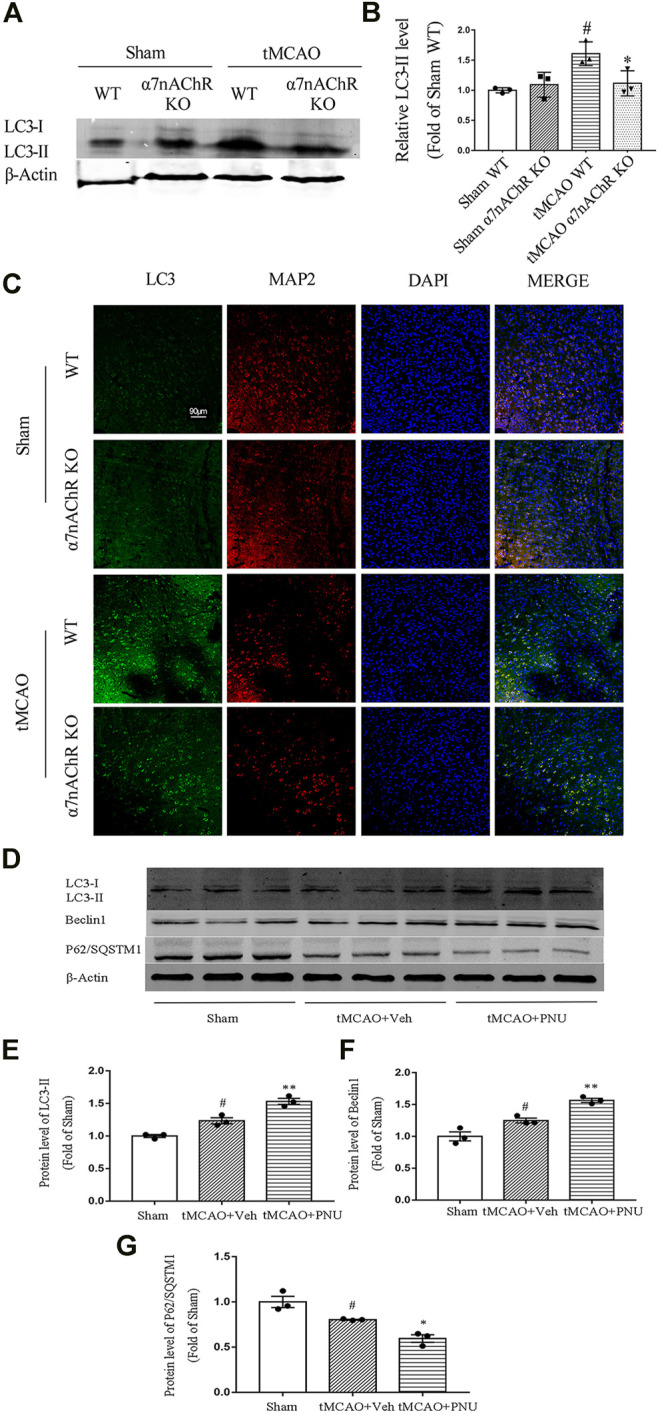
α7nAChR knockout decreases autophagy in neurons following tMCAO. Wild-type (WT) mice, α7nAChR knockout (KO) mice, and C57BL/6 mice were subjected to sham or tMCAO surgery. Brains were collected at 24 h after tMCAO surgery. The penumbral zone of the brains was isolated, and protein levels of LC3, Beclin1, and P62/SQSTM1 were detected by Western blot. Paraffin-embedded coronal brain sections were prepared for immunofluorescence staining of LC3, MAP2, and DAPI. **(A,B)** LC3 level was increased after tMCAO in WT mice compared with that in the sham group; however, α7nAChR KO mice alleviated this effect. *n* = 3 for each group. ^#^ *p* < 0.05 vs*.* Sham WT, *p < 0.05 vs*.* MCAO WT. **(C)** Representative images of immunofluorescence microscopy. Scale bar, 90 μm. **(D–G)** Protein expression of LC3, Beclin1, and P62/SQSTM1 was detected by Western blot. *n* = 3 per group. ^#^ *p* < 0.05 vs*.* Sham; *p < 0.05 vs*.* MCAO + Veh; and **p < 0.01 *vs.* MCAO + Veh.

Previously, our lab found that activation of α7nAChR could induce autophagy to protect against EAE. Hereby, we wanted to know whether the endogenous activator of α7nAChR could regulate autophagy in ischemic stroke mice. tMCAO operation was performed on WT and α7nAChR KO mice. At 24 h after the operation, brains were collected and autophagy-related protein LC3 level (LC3-II) in the penumbral zone was detected using Western blot. The results revealed that the LC3-II level was increased after tMCAO operation compared with that in the sham group, which was consistent with other studies (Wang et al., 2012). Moreover, we found that after tMCAO operation, the LC3-II level in α7nAChR KO mice was significantly decreased compared with that in WT mice (Figures 3A,B), suggesting that activating α7nAChR could enhance autophagy.

Our previous studies demonstrated that activation of α7nAChR could promote autophagy in microglia which plays a positive role in EAE mice. In this study, we try to clarify the role of α7nAChR in neuron autophagy after ischemic stroke. Thus, we detected autophagy-related protein LC3 neurons (green) and neuronal marker MAP2 (red) in neurons using co-immunofluorescence. We found that after tMCAO operation, the LC3 level in neurons was increased. However, the LC3 level in neurons in α7nAChR KO mice was significantly decreased when compared with that in the WT group (Figure 3C). Collectively, these results demonstrated that α7nAChR could promote autophagy in neurons.

Besides, we also detected the activation of α7nAChR by PNU282987 in promoting autophagy in tMCAO C57BL/6 mice. From the result, we can find out that tMCAO resulted in enhanced autophagy, while activation by PNU282987 could further enhance autophagy (Figures 3D,E–G). The results were consistent with the above in knockout mice.

### Activating α7nAChR Promotes Autophagy in Neurons Exposed to OGD/R

**FIGURE 4:**
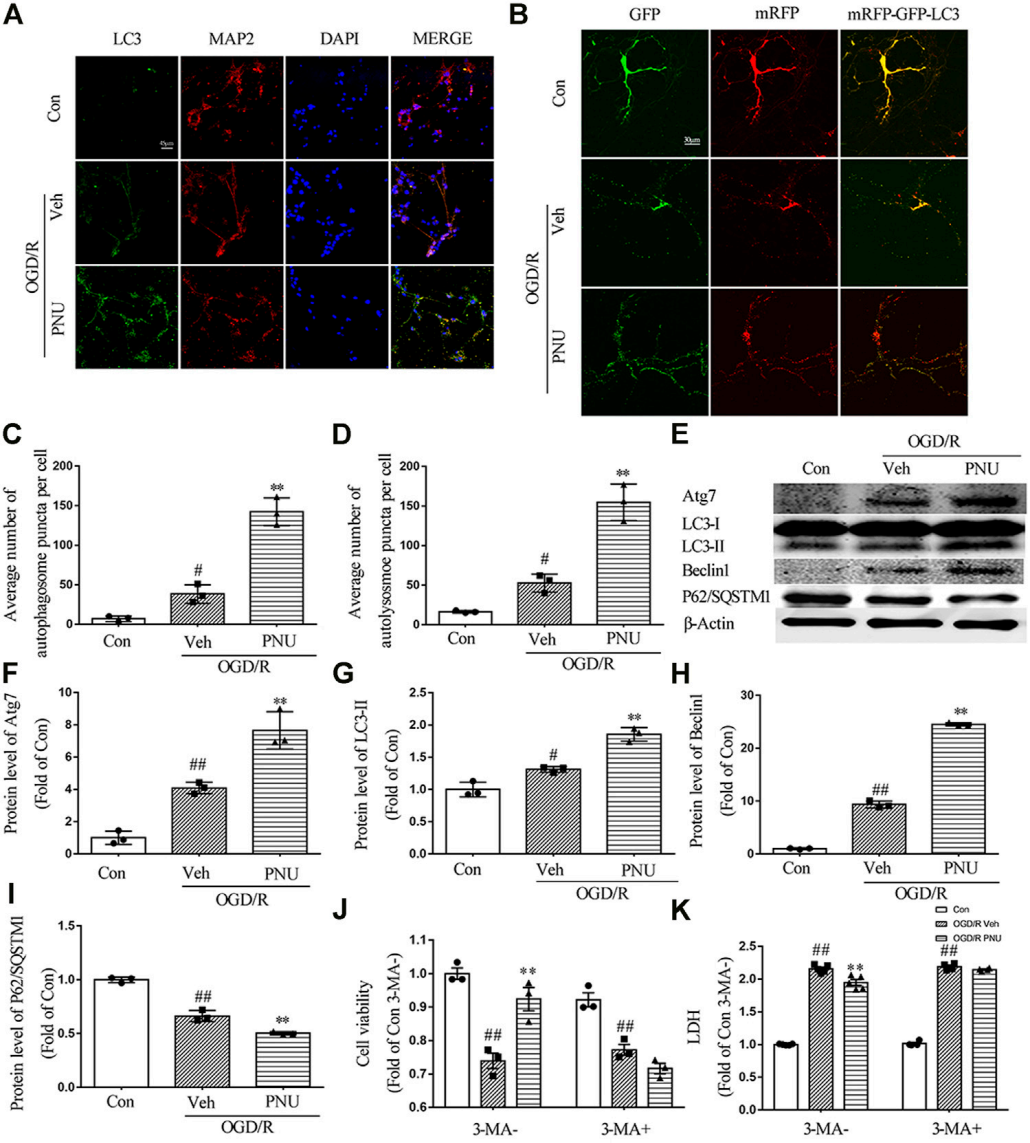
α7nAChR activation enhances autophagy in neurons upon OGD/R. Primary cortical neurons were treated with vehicle or PNU282987 (100 μM) for 2 h before OGD/R. The level of autophagy was detected at 24 h after OGD/R. **(A)** Representative images of immunofluorescence staining of LC3, MAP2, and DAPI. Scale bar, 45 μm. **(B)** Representative images of autophagic flux. Lentivirus-harboring tandem monomeric mRFP-GFP-LC3 were transfected into neurons. Yellow puncta represent autophagosomes, and red puncta represent autolysosomes. PNU282987 enhanced autophagic flux compared to OGD/R groups. Scale bar, 30 μm. **(C)** Quantification of autophagosome puncta. *n* = 3 per group. **(D)** Quantification of autolysosome puncta. *n* = 3 per group. **(E–I)** Autophagy-related proteins were detected by Western blot. *n* = 3 per group. **(J)** Pretreatment with 3-MA (1 mM, an autophagy inhibitor) significantly inhibited the effect of PNU282987 on cell viability. *n* = 3 for each group. **(K)** Pretreatment with 3-MA (1 mM, an autophagy inhibitor) significantly inhibited the effect of PNU282987 on LDH release. *n* = 5 for each group. ^#^ *p* < 0.05 vs. con; ^##^ *p* < 0.01 vs. con; and **p < 0.01 vs. OGD/R veh.

To determine whether autophagy is involved in the protective effect of α7nAChR on cultured neurons, cultured primary cortical neurons were treated with vehicle or 100 μM PNU282987 before OGD/R exposure. We found that OGD/R exposure increased the numbers of LC3 positive neurons. PNU282987 treatment led to a significant increase of LC3 positive neurons compared with the vehicle group (Figure 4A). To further detect the influence of activating α7nAChR on autophagic flux, a dynamic process, lentivirus-harboring tandem monomeric mRFP-GFP-LC3 was transfected into cultured primary cortical neurons. As the GFP signal is sensitive to the acid environment in lysosomes and is easily eliminated while mRFP is more stable, autophagosomes will show yellow puncta and autolysosomes will show red puncta (Klionsky et al., 2016). The results showed that OGD/R exposure increased the numbers of both autophagosomes and autolysosomes, indicating an enhanced autophagic flux. Compared with the vehicle group, PNU282987 treatment further increased the numbers of autophagosomes and autolysosomes (Figures 4B–D).

Although quantification of LC3 by immunofluorescence may be more sensitive and quantitative than monitoring LC3-II by Western blot, the guideline recommends both assays and comparing the two sets of results (Klionsky et al., 2016). Thus we detected autophagy-related proteins by Western blot. OGD/R exposure increased the expression of Atg7, LC3-II, and Beclin 1. PNU282987 treatment further increased the level of Atg7, LC3-II, and Beclin 1. Besides, the expression of P62/SQSTM1 was reduced after OGD/R, and PNU282987 treatment could further decrease the level of P62/SQSTM1 (Figures 4E–I). Taken together, activation of α7nAChR by PNU282987 could promote autophagy in neurons exposed to OGD/R.

To further determine whether autophagy is involved in the beneficial effect of α7nAChR on neurons, the cultured primary cortical neurons were pretreated with PNU282987 with or without 3-MA (1 mM, an autophagy inhibitor) before OGD/R exposure. The result showed that 3-MA significantly attenuated the effect of PNU282987 on cell viability and LDH release (Figures 4J,K), suggesting that autophagy is involved in the beneficial effects of α7nAChR activation on neurons.

### AMPK-mTOR Pathway is Involved in the Protection of α7nAChR Activation

**FIGURE 5:**
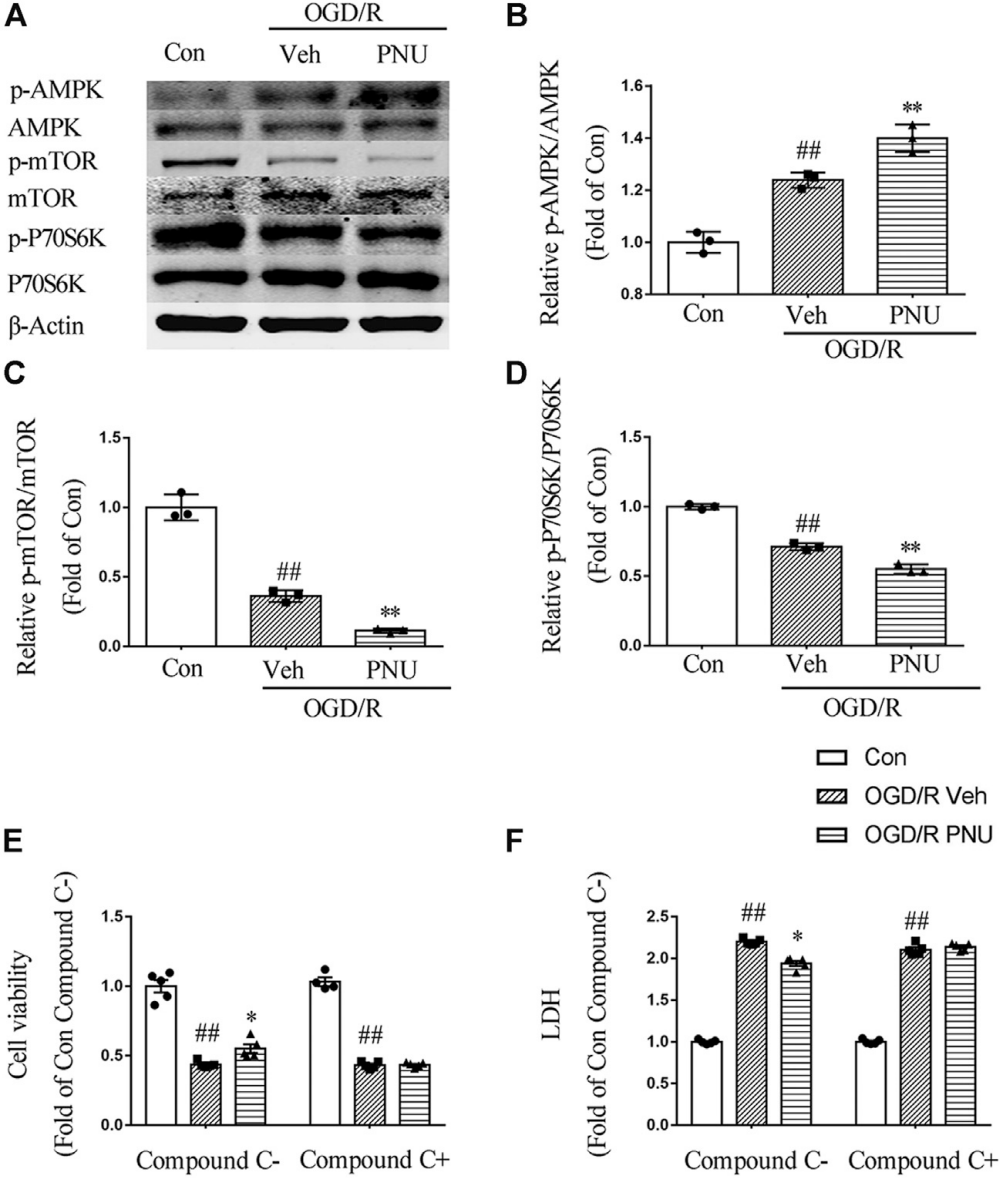
AMPK-mTOR pathway is involved in the neuroprotection of α7nAChR activation. Primary cortical neurons were treated with or without PNU282987 100 μM for 2 h before OGD/R exposure. **(A–D)** The expression and phosphorylation of AMPK, mTOR, and P70S6K were detected by Western blot. OGD/R exposure increased the level of p-AMPK and decreased the levels of p-mTOR and p-P70S6K. PNU282987 treatment further enhanced the level of p-AMPK and reduced the levels of p-mTOR and p-P70S6K. *n* = 3 for each group. **(E, F)** In addition, compound C (5 μM), an AMPK inhibitor, significantly inhibited the effects of PNU282987 treatment on cell viability and LDH release. *n* = 5 for each group. ^##^ *p* < 0.01 vs. con; *p < 0.05 vs. OGD/R veh; and **p < 0.01 vs*.* OGD/R veh.

To explore the signaling pathway involved in the α7nAChR-mediated autophagy and neuroprotective effects, we detected the expression and phosphorylation of AMPK, mTOR, and P70S6K in primary cortical neurons pretreated with vehicle or PNU282987 before OGD/R exposure. The results showed that OGD/R exposure increased the level of p-AMPK. PNU282987 treatment further enhanced the level of p-AMPK. In the meantime, the levels of p-mTOR and p-P70S6K were significantly decreased after OGD/R exposure, and PNU282987 treatment further reduced the levels of p-mTOR and p-P70S6K (Figures 5A–D). In addition, compound C (5 μM), an AMPK inhibitor, significantly inhibited the effects of PNU282987 treatment on cell viability and LDH release (Figures 5E,F). Collectively, these data suggested that AMPK-mTOR-P70S6K may play a role in the neuroprotective effect of activating α7nAChR.

## Discussion

The protective effect of α7nAChR on several central nervous system diseases, such as ischemic stroke, hemorrhagic stroke, multiple sclerosis, and Alzheimer’s disease, has been demonstrated in substantial studies (Duris et al., 2011; Cheng et al., 2013; Guo et al., 2017; Shao et al., 2017). Our lab also found that activating α7nAChR protects against hypertension and crushing syndrome through the cholinergic anti-inflammatory pathway (Li et al., 2011; Xu et al., 2016). To investigate the role of α7nAChR in ischemic stroke, we applied the tMCAO model. However, the previous studies using the tMCAO model claimed a variety of occlusion times, from 30 min to 2 h (Wang et al., 2012; Yan et al., 2016). The infarct volume varies largely because it can be influenced by a series of factors like the operation experience of the researcher, temperature, diameter of embolus, and so on. Here, we applied 2 h as our previous studies claimed (Liu et al., 2012a; Liu et al., 2013). Our *in vivo* data showed that expression of α7nAChR was decreased upon the tMCAO model. However, it was uncertain whether α7nAChR was playing a protective role or a deteriorative role in ischemic stroke. To answer this question, we used α7nAChR KO mice to observe the effect. The result showed that compared to the WT mice, α7nAChR KO deteriorated ischemic stroke. Then, we tried to figure out how α7nAChR affects prognosis of ischemic stroke. The result showed that compared to the WT mice, the neuronal autophagy level decreased in α7nAChR KO mice after tMCAO operation. Some studies reported that early enhanced autophagy was beneficial to the prognosis of tMCAO. So we assumed that poor prognosis after α7nAChR knockout related to decreased autophagy. To further demonstrate this hypothesis, we cultured primary neurons *in vitro* and found that activation of α7nAChR by PNU282987 could inhibit apoptosis and induce autophagy to protect neurons from OGD/R condition. Results also revealed that the AMPK-mTOR-p70S6K signaling pathway was involved in the α7nAChR-mediated neuroprotection. Thus, in this study, we revealed that activating α7nAChR plays a protective role in ischemic stroke by regulating neuronal autophagy and apoptosis through the AMPK-mTOR-p70S6K signaling pathway.

As a subtype of nicotinic acetylcholine receptors, α7nAChR is expressed on a variety of cell types in the central nervous system, such as neurons, astrocytes, and microglia (Liu et al., 2012b). Our lab found that activation of α7nAChR on microglia could lead to anti-inflammation effect, which contributes to relief of multiple sclerosis (Shao et al., 2017). Recently, Han et al. (2014b) found that activation of α7nAChR reduced ischemic stroke through inhibiting the production of pro-inflammatory and oxidative stress mediators in microglia, and Zou et al. (2017) found that activation of α7nAChR could also reduce brain edema. In this study, we used α7nAChR knockout mice to prove that the presence of α7nAChR could reduce the infarct volume of ischemic stroke and improve neurological function, which was consistent with previous studies. Our *in vitro* data demonstrated the protection of PNU282987 on neurons through increase of cell viability, reduction of LDH release, and reduced TUNEL positive cells upon OGD/R exposure. Furthermore, the results showed that activation of α7nAChR could decrease apoptosis, which strongly suggested that α7nAChR might protect neurons under ischemic stroke. The results showed that the level of α7nAChR decreased after ischemic stroke *in vivo*, and the level of α7nAChR on neurons decreased after OGD/R *in vitro* as well, which may deteriorate the outcome of ischemic stroke.

Autophagy is a common process in a variety of cells, wherein damaged organelles and misfolded or unused proteins can be degraded into metabolic components for recycling to maintain cellular homeostasis. It is widely accepted that lack of energy can enhance autophagy. However, whether enhanced autophagy is beneficial for cells is still controversial. Some studies demonstrated that autophagy has a protective effect on ischemic stroke, while others discovered the opposite effect. Jiang and Wang found that suppression of autophagy in microglia significantly reduced the OGD/R-induced inflammatory response (Jiang et al., 2018). However, Zhu and Shui found that increased autophagy degradation contributed to the neuroprotection in human neuroblastoma cell lines (SH-SY5Y) (Zhu et al., 2017). In our *in vitro* study, we used PNU282987 to induce autophagy, and from the results of increased cell viability of the CCK-8 assay and reduced cytotoxicity of LDH release, we found that autophagy could rescue neurons from OGD/R-induced neuronal injury. The differences between our results and those of previous studies may be owing to different cell types tested, different agents used, and different time points or periods for treatment.

The relationship between α7nAChR and autophagy has been revealed by Jeong and Park (2015). In their study, they found that melatonin-mediated autophagy relies on the activation of α7nAChR, which plays a pivotal role in neuroprotection of prion-mediated mitochondrial neurotoxicity. They used PrP (106–126) to induce the neurotoxicity on SH-SY5Y cells to demonstrate. In this study, we used PNU282987, a selective agonist for α7nAChR, to demonstrate that the activation of α7nAChR could enhance autophagy in neurons. To determine whether PNU282987 protects neurons from OGD/R exposure through autophagy, 3-MA was used, which could block autophagosome formation via inhibiting class III PI3K. The result showed that after the use of 3-MA, the protective effect of PNU282987 on neurons disappeared. However, there is a limitation to drawing a solid conclusion. It is better inhibit lysosomal to exclude the mis-interruption of lysosomal dysfunction. Taking the results together, we draw a conclusion with caution that the protective effect of PNU282987 on neurons relies on the enhancing of autophagy via activating α7nAChR.

In this study, we proved that activation of α7nAChR could induce autophagy in neurons, which happens partly through the AMPK-mTOR-p70S6K signaling pathway. However, it is not the only signaling pathway involved in α7nAChR-induced autophagy. Previous studies showed other signaling pathways involved in α7nAChR-mediated neuronal protection. For instance, Marrero and Bencherif (2009) reported that the JAK2-STAT3-NF-κB signaling cascade was involved in the α7nAChR-mediated anti-apoptosis effect in PC12 cells. Besides, our previous study also found that JAK2-STAT3 was involved in the protective effect of α7nAChR in crush syndrome (Xu et al., 2016). It was also reported by Parada and Egea that activation of α7nAChR could protect the brain from ischemic conditions through the α7nAChR/Nrf2/HO-1 axis in microglia, which belongs to the cholinergic anti-inflammation pathway (Parada et al., 2013). To sum up, neuronal protection effects of α7nAChR involve several signaling pathways. To induce autophagy in neurons via AMPK-mTOR-p70S6K could partly account for the protective effect of α7nAChR. Therefore, further studies should be conducted to reveal whether these proved signaling pathways activated by α7nAChR have crosstalk.

In conclusion, our findings demonstrate the neuroprotective effect of α7nAChR activation via enhancing autophagy and mobilizing the AMPK-mTOR-p70S6K signaling pathway after ischemia. The study not only elucidates the exact molecular mechanism of α7nAChR in ischemic stroke but also contributes to the development of new treatment targeting α7nAChR, hoping to promote the new drug discovery for ischemic stroke.

## Data Availability

The raw data supporting the conclusions of this article will be made available by the authors, without undue reservation.
